# Variation in Soil Organic Carbon Along an Altitudinal Gradient Across Different Aspects in the Timberline Zone of the Western Himalaya, India

**DOI:** 10.3390/plants15182775

**Published:** 2026-09-10

**Authors:** Renu Rawal, Ankur Sharma, Gaurav Mishra, Tanay Barman, Ravi K. Chaturvedi, Lalit M. Tewari

**Affiliations:** 1 Department of Botany, Pt. B.D. Pandey Campus Bageshwar, Soban Singh Jeena University Almora, Almora 263601, Uttarakhand, India; renu.rawal93@gmail.com; 2 G.B. Pant National Institute of Himalayan Environment (GBP-NIHE), Kosi-Katarmal, Almora 263643, Uttarakhand, India; 3 Forest Ecology & Climate Change Division, ICFRE-Forest Research Institute, P.O. New Forest, Dehradun 248006, Uttarakhand, India; ankursharma.fri@gmail.com; 4 Centre of Excellence on Sustainable Land Management, Indian Council of Forestry Research and Education, P.O. New Forest, Dehradun 248006, Uttarakhand, India; gaurav.mishra215@gmail.com; 5 Center for Integrative Conservation, Xishuangbanna Tropical Botanical Garden, Chinese Academy of Sciences, Menglun, Mengla, Jinghong 666303, China; 6 Department of Botany, DSB Campus, Kumaun University, Nainital 263001, Uttarakhand, India

**Keywords:** topographic controls, alpine ecotone, digital soil mapping, microclimate, edaphic properties

## Abstract

The timberline region of the Western Himalaya is a crucial ecological transition area highly sensitive to climate changes, which significantly influence vegetation patterns, soil formation, and carbon dynamics. This study aimed to investigate the spatial and altitudinal changes in soil organic carbon (SOC) across different topographic orientations and to evaluate machine-learning models for spatial SOC prediction in the timberline ecotone (2100–3300 m) of the Kedarnath Wildlife Sanctuary. Through systematic random sampling across 100 m elevation bands, composite soil samples were collected from three aspects (North-East, South-West, and North-West) at two depths (0–15 cm and 15–30 cm) and analyzed alongside topographic, spectral, and climatic covariates. Results indicated that SOC trends varied significantly by aspect; the North-East aspect exhibited a considerable increase in SOC with elevation, while the North-West and South-West sides responded differently. Furthermore, Digital Soil Mapping using the Random Forest (RF) model outperformed Support Vector Machine and XGBoost, explaining 62% of surface and 74% of subsurface SOC variability. In conclusion, aspect-induced microclimatic gradients play a major role in controlling soil characteristics near the Himalayan timberline, and machine-learning models like RF can successfully capture these complex spatial patterns. Consequently, it is recommended that the established baseline SOC maps be utilized as reference points for future climate-change monitoring, carbon accounting, and directing conservation strategies in high-altitude forests.

## 1. Introduction

The Himalayan timberline is one of the most fragile ecotones, where soil processes and vegetation are highly responsive to climate change [1]. Soil properties including moisture, bulk density, pH, texture, and nutrients are important in controlling plant dynamics and ecosystem functioning. Different microclimates that affect soil formation, decomposition, and nutrient cycling are created by variations along altitudinal gradients and aspects. Predicting soil degradation, treeline movements, and ecosystem resilience under climate change requires an understanding of these processes [2]. At the timberline, the physical characteristics of the soil, such as texture, bulk density, and porosity, vary predictably with elevation and aspect [3]. These differences have a significant impact on root development and water retention. Higher altitudes often have colder soils that accumulate more organic matter (where plant cover is present), retain more moisture throughout the growing season, and have a lower bulk density than lower-elevation soils that have been disturbed by human activities or have undergone more decomposition [4,5]. Despite their high height, steep, wind-exposed ridges may have shallow, stony soils with poor water-holding ability due to local rockiness, slope, and snowfall regime. Throughout the timberline ecotone, these micro-site disparities result in fine-scale mosaics of tree establishment and development [6].

Nutrient cycle and soil chemistry are equally significant. Altitude and plant cover frequently affect soil pH, SOC, accessible nitrogen, and phosphorus. In many Himalayan transects, SOC and microbial biomass can increase with altitude where woody cover contributes litter inputs and slower decomposition due to lower temperatures; yet, nutrient mineralization rates often decline with elevation because of cooler soils and shorter seasons, potentially limiting seedling growth even when levels of organic matter are relatively high [7]. Therefore, whether microsites are conducive to the recruitment and persistence of treeline species depends on the equilibrium between litter inputs, decomposition, and nutrient release.

Microclimatic gradients that affect soil temperature, moisture content, and snow persistence are imposed by aspect (north vs. south slopes) and local topography. In comparison to north-facing slopes, south-facing slopes in the Himalayan area usually get more solar radiation, have warmer, drier soils, and exhibit quicker decomposition and lower soil moisture [8]. Within short distances, these aspect-driven variations frequently result in asymmetric treeline placements and disparate species compositions [9]. Aspect can have an impact on the importance of establishing the growth window for seedlings, which is a crucial factor in determining long-term treeline migration as it also affects snow accumulation and melt timing.

Treelines are predicted to move upslope in many temperate mountain ranges because of continuous climate change, although the rate and extent of this movement are influenced by the local microclimate and soil characteristics. In the near term, warmer soils may promote tree growth and seedling survival by increasing mineralization and nutrient availability. On the other hand, despite favorable mean temperatures, warming can change the time and depth of snowfall, expose seedlings to late frost episodes, or increase evapotranspiration and drought stress on south-facing aspects, all of which are factors that could delay growth. The necessity of combining soil and microtopographic data for forecasting treeline dynamics is highlighted by recent Himalayan studies that show both upslope gains in certain areas and intricate, site-specific responses elsewhere [10].

From a process standpoint, three interrelated soil-mediated mechanisms are particularly significant for Himalayan timberlines: (1) water availability for seedlings (determined by texture, porosity, and snowmelt patterns); (2) nutrient supply through litter decomposition and microbial activity (sensitive to soil temperature and moisture); and (3) mechanical and rooting constraints imposed by shallow or rocky soils (which limit root anchorage and access to water). By changing plant cover, compaction, and organic inputs, disturbances including grazing, fuelwood harvest, and landslides further modify these systems. Monitoring soil moisture regimes, SOC pools, bulk density, and important nutrients over elevation and aspect gradients provides crucial information on ecosystem resilience and expected treeline trajectories for management and prediction [11,12].

Digital soil mapping (DSM) integrates field observations with spatial environmental data and machine-learning algorithms to generate high-resolution soil property maps [13]. We applied DSM methods to create prediction maps of SOC distribution across the timberline zone. Unlike traditional soil survey methods, which rely on labor-intensive field sampling and expert interpretation, DSM combines soil observations with topographical features, climatic factors, vegetation indices, and remote sensing datasets to provide repeatable, high-resolution soil information. The theoretical underpinning of DSM is based on the SCORPAN model, which ties soil variation to elements such as climate, organisms, terrain, parent material, age, and spatial location [14]. Advances in processing power, machine-learning algorithms, and satellite-data availability have hastened the use of DSM, enabling more accurate soil forecasts across varied landscapes, including hilly and data-poor locations [15].

Understanding soil–vegetation interactions close to the timberline is essential for conservation planning, carbon budgeting, and local livelihoods that depend on mountain forests. Despite the significance of SOC and the availability of advanced interpolation techniques like DSM, limited information is available on how altitudinal and aspect variation in the Timberline Zone of the Western Himalaya influence SOC distribution. While existing research [16,17,18,19] has explored broad soil–vegetation interactions and treeline dynamics in the Himalayas, these studies often overlook the combined microclimatic effects of altitude and slope aspect specifically on soil organic carbon (SOC) distribution. To address this spatial and thematic limitation, this study focuses explicitly on the altitudinal and aspect-wise variation in SOC within the timberline zone of the Western Himalaya. Therefore, the present study aimed to (i) assess the effects of altitude and aspect on SOC across the Timberline Zone of the Western Himalaya and (ii) review the spatial prediction of SOC using machine-learning models. The results are anticipated to offer important perspectives for land management and climate-resilient conservation approaches in the Timberline Zone of the Western Himalaya.

## 2. Material and Methods

### 2.1. Study Site

**Figure 1:**
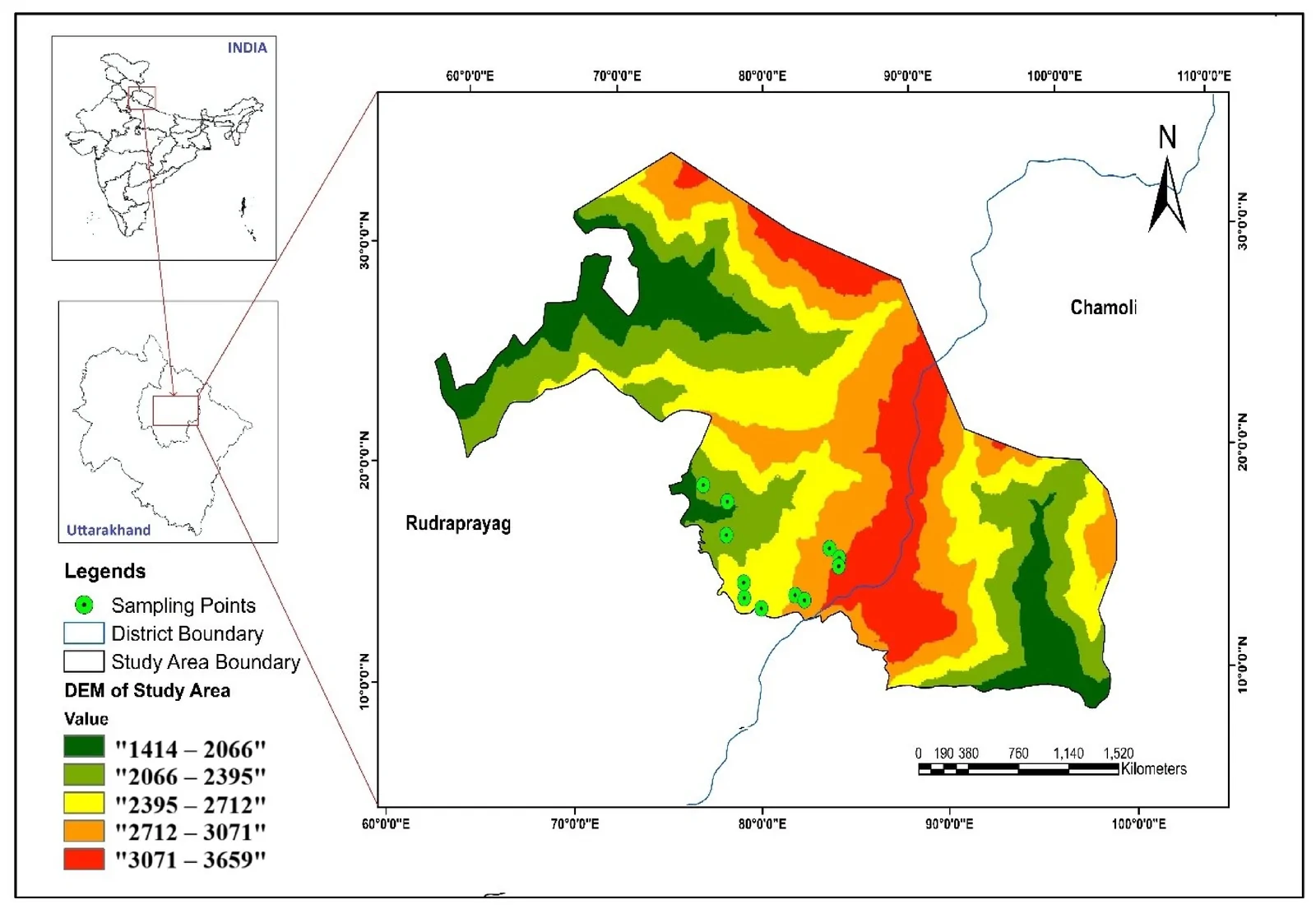
Geographical location of the study site in the Kedarnath Wildlife Sanctuary, showing elevational transects and aspect-wise sampling layout.

The intensively surveyed site comes under Kedarnath Wildlife Sanctuary (KWS), broadly known as Chopta-Tungnath region (30°30′03.0″ to 30°29′23.8″ N and 79°09′52.8″ to 79°12′42.3″ E) of Uttarakhand, Western Himalaya (Figure 1). The present study covers an elevation range from about 2100 to 3300 m asl., covering three available aspects in the region: North-West (NW), South-West (SW) and North-East (NE). The study is concerned with an analysis of change in soil organic carbon (SOC) characteristics across three aspects within 100 m elevation belts from lower closed-canopy forests (2100 m) to sub-alpine (Timberline) zones (3300 m) [20]. The forest communities within this gradient ranged from lower temperate forests, which dominated, to sub-alpine zones, and gradually transitioning into alpine meadows beyond the treeline [21,22,23,24,25]. The lower elevations were dominated by evergreen oak species (*Quercus leucotrichophora*), *Machilus duthiei*, *Rhododendron arboreum* and the middle elevations were dominated by brown oak (*Q. semecarpifolia*), which was occasionally mixed with *Abies pindrow* and *R. arboreum*. However, the higher elevations were dominated by *Q. semecarpifolia*, *A. spectabilis* and *R. campanulatum* [25]. The mean annual temperature in the study site ranged between 5.6 °C at 3360 m and 17.6 °C at 1500 m elevation in North-West aspect, and in South-East aspect ranged between 6.0 °C at 3680 m and 17.2 °C at 1600 m elevation. The mean annual rainfall in North-West aspect ranged between 1714.0 mm at 1500 m and 2953.0 mm at 3360 m elevation, while in South-East aspect it ranged between 2209.0 mm at 2100 m and 2770 mm at 3100 m elevation. The soil is predominantly hill soil that varies in color from dark brown to light brown, greyish-brown, or pale yellow, depending on local vegetative cover and altitude. The texture ranges from sandy loam to finer loamy compositions, which are heavily influenced by the local slope and depth of the terrain, and are generally acidic, with pH ranging from 4 to 6 [26,27].

### 2.2. Soil-Sampling Approach and Analysis

**Figure 2:**
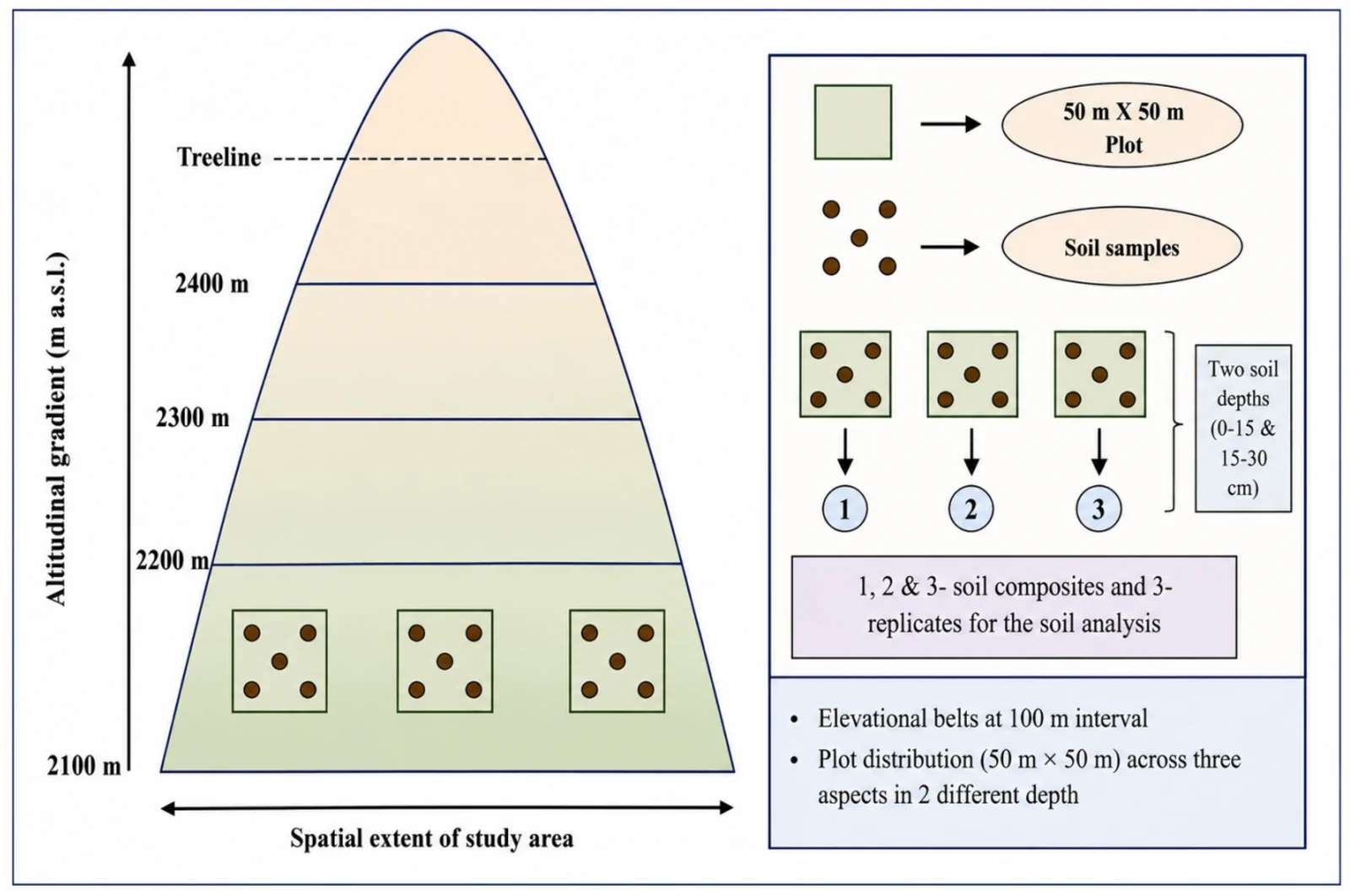
Diagrammatic representation of sampling method along an altitudinal gradient in the Kedarnath Wildlife Sanctuary (generated with the help of Artificial Intelligence).

Three representative elevation transects (from 2100 m to 3300 m) in three different aspects (SW, NE and NW) in the study site were investigated to obtain generalized compositional patterns of soil organic carbon. In general, soil samples were collected each year from April to mid-June, from 2021 to 2023. The study transects (aspects) were investigated by systematically dividing each transect into 100 m elevational belts. Within each 100 m elevation belt, soil properties were investigated using random sampling method. This approach represents a systematic random sampling. For an adequate representation of a given plot (50 m × 50 m), samples were collected from 5 different locations, generally one from the center and four from the corners, at two soil depths (upper 0–15 cm and lower 15–30 cm). Later, homogenized composite soil samples were prepared after thoroughly mixing the soil from similar depths together and, after that, from the composite soil; around 100 gm soil samples from each plot were brought to the laboratory in air-tight polythene bags for analysis. A soil auger is a tool used to dig holes in the ground or collect soil samples. The samples were air-dried until a constant weight was achieved in shade and then filtered through 2 mm sieve. Dried samples were analyzed for SOC using the methodology given by [28]. Diagrammatic representation of sampling method along an altitudinal gradient in the Kedarnath Wildlife Sanctuary has been provided in Figure 2.

### 2.3. Environmental Covariates

A Digital Elevation Model (DEM) with a resolution of 90 m was produced using data from the Shuttle Radar Topographic Mission (SRTM). Table 1 shows the primary and secondary derivatives of the DEM that were obtained using the Saga-GIS 6.3.0 version. These derivatives included elevation (Elev), slope (Slp), analytical hill shade (AH), aspect (Asp), valley depth (VD), channel network base level (CNBL) and distance (CND), relative slope position (RSP), plan curvature (PlaC), profile curvature (PrC), topographic wetness index (TWI), topographic ruggedness index (TRI), topographic position index (TPI), LS factor, multi-resolution index of valley bottom flatness (MRVBF), and total catchment area (TCA). Normalized difference vegetation index (NDVI) and enhanced vegetation index (EVI) were employed as covariates in addition to DEM variables to estimate soil depth. The world’s raster data on bioclimatic variables at a resolution of 1 km were obtained from http://worldclim.org/current (Access date: 1 June 2026), and the corresponding research area grids were then derived from these global grids. In the study of digital soil mapping, all 19 bioclimatic variables were chosen as covariates. Ref. [29] provide a thorough explanation of these bioclimatic factors. MODIS, WorldClim, and DEM data are widely used for soil-depth prediction due to their broad coverage and availability. However, these datasets often have coarse spatial resolutions, which may capture variations in heterogeneous landscapes on a regional scale. The environmental parameters were extracted at every sampling point to predict the soil depth. To create a common grid of 90 × 90 m, all auxiliary variables from the three source databases were aggregated (average resampling) or disaggregated (bilinear resampling) and used as auxiliary variables for the creation of quantitative spatial models. The coordinates of all the data were translated to UTM WGS84 Zone 46 N and were in raster format.

### 2.4. Statistical Analysis

The relationship of soil organic carbon to elevation was described using liner regression model, in STATISTICA.lnk, where the value of R^2^ was explained (the greater the value of R^2^, the stronger the relationship between variables x and y, independent and dependent, respectively, where y is the variance) for each parameter; *p*-value was tested and explained at 95% significance level: *p*- value less than 0.05 (significant) and 0.01 (highly significant).

The significant difference between the soil depths for each soil property was determined by two tailed *t*-tests, applied as both dependent variables, and depicted by box whisker plot, which explains the significant (*p* < 0.05 significant and *p* < 0.01 highly significant) and non-significant (*p* > 0.05) difference between the mean of both soil depths (upper 0–15 cm and lower 15–30 cm).

## 3. Results

### 3.1. Pattern of Soil Organic Carbon Along Elevation Gradient Across Three Aspects

**Figure 3:**
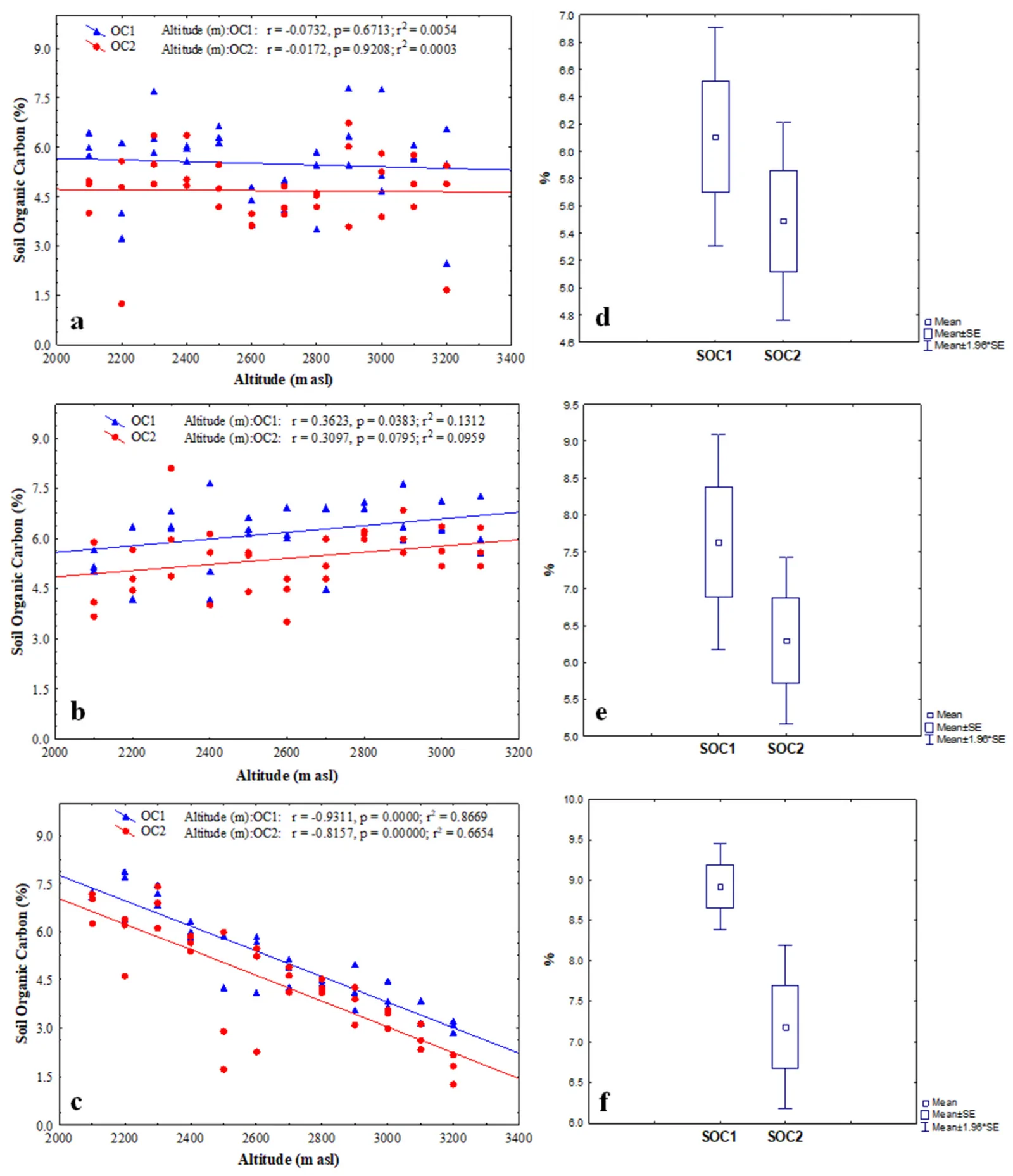
Trends of soil organic carbon (SOC) along the elevation gradient: (**a**) SW aspect (both depths ^ns^), (**b**) NE aspect (upper depth * and lower depth ^ns^), (**c**) NW aspect (both depths **); and Box whisker plots of *t*-test applied on SOC for two soil depths: (**d**) SW aspect ^ns^, (**e**) NE aspect * and (**f**) NW aspect **. (* significant at *p* < 0.05; ** highly significant at *p* < 0.01; and ^ns^ non-significant).

Along the elevation gradient, the soil organic carbon content tended to decrease significantly in the NW aspect (upper depth: R^2^ = 0.87; *p* < 0.01 and lower depth: R^2^ = 0.67; *p* < 0.01; Figure 3c) and in the NE aspect it tended to increase significantly for the upper depth (R^2^ = 0.13; *p* < 0.05; Figure 3b), while for the lower depth of the NE aspect, and for both depths of the SW aspect, it did not show significant correlation with the elevation (Figure 3a,b). The soil organic carbon ranged between 4.28 and 6.60% at the upper depth and 3.75 and 5.59% at the lower depth of the SW aspect; in the NE aspect it ranged between 5.28 and 6.74% at the upper depth and 4.26 and 6.32% at the lower depth; and in the NW aspect it ranged between 3.06 and 7.32% at the upper depth and 1.75 and 6.82% at the lower depth (Table 2). Further considering the soil organic carbon (SOC) depth-wise relationship, it showed significantly greater content in the upper soil depth in the NE aspect (*p* < 0.05; Figure 3e) and the NW aspect (*p* < 0.01; Figure 3f), while the SW depth-wise relationship was non-significant (Figure 3d).

### 3.2. Correlation Between Variables

**Figure 4:**
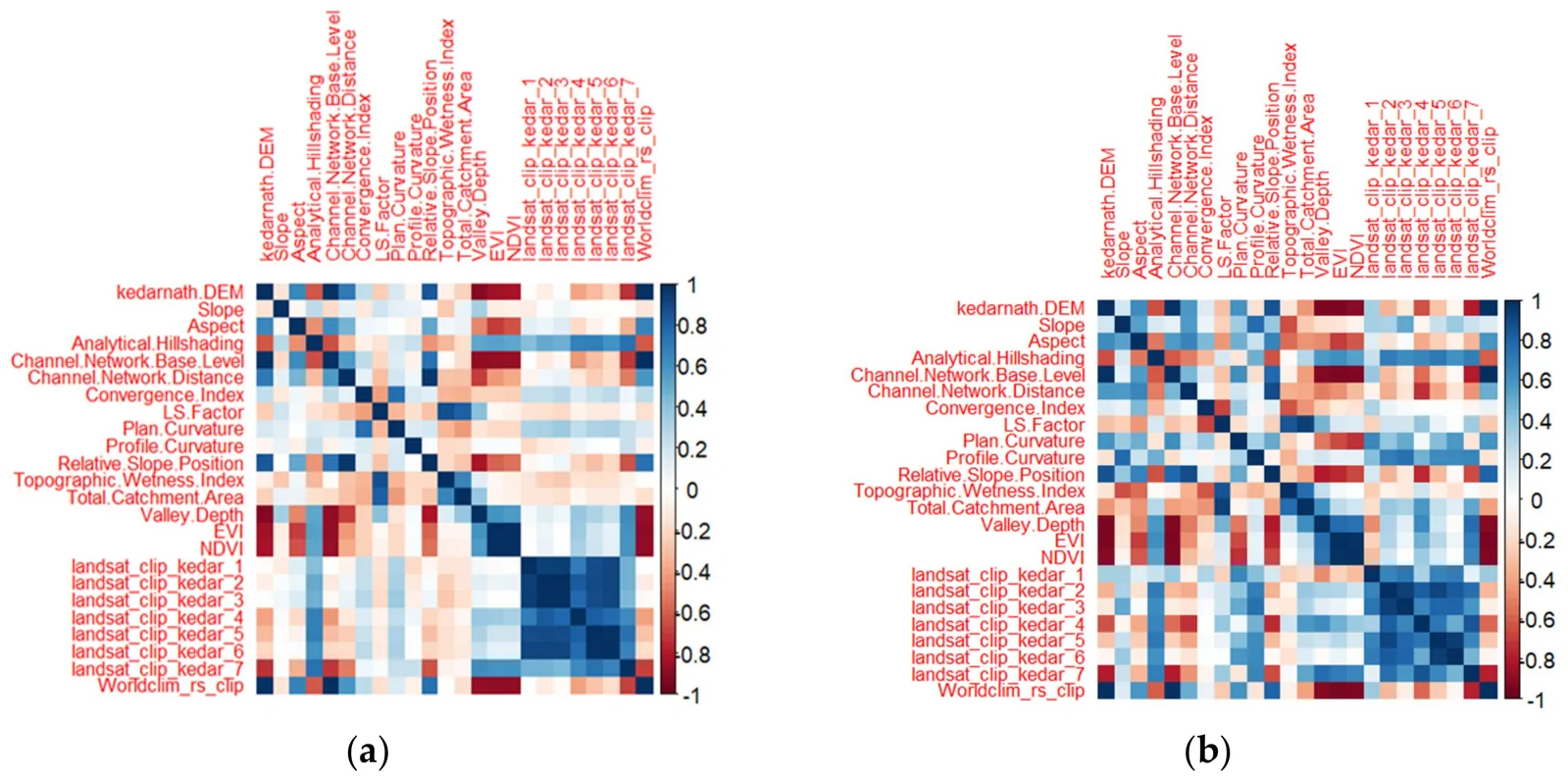
(**a**,**b**) Correlation analysis between covariates and soil depth (**a**) 0–15 cm and (**b**) 15–30 cm depth.

The correlation heatmap for the 0–15 cm layer shows distinct covariate groups between terrain derivatives, Landsat spectral bands, vegetation indices, and climatic factors. Terrain-based predictors (DEM, slope, aspect, curvature parameters, TWI, RSP, LS factor, channel network features, and catchment metrics) exhibited moderate to high internal correlations due to their common geomorphological basis. There was a high positive correlation between slope and LS-factor, consistent with their mathematical dependency. Negative correlations between base level and channel network distance and valley depth and terrain wetness indicated topographic control over soil-moisture distribution. Spectral characteristics (Landsat bands 1–7) formed a unique cluster with substantial internal correlations. The strongest cohesiveness was observed in shortwave infrared (bands 5–7), consistent with their sensitivity to surface roughness, mineral composition, and soil wetness. Vegetation indices (EVI and NDVI) showed substantial relationships with red-NIR-SWIR spectral bands, indicating high vegetation signal stability in the surface layer. Limited climatic variability across the relatively small geographical area was shown by the weaker correlations of Worldclim climatic variables with both terrain and spectral predictors. At the 15–30 cm depth, the correlation heatmap shows a generally comparable clustering structure for the surface layer, though with noticeable changes in the intensity and direction of relationships. At this depth, the relationship between terrain features and spectral and vegetation indices weakened, though they still maintained substantial intercorrelation. This decline indicates that vegetation and surface reflectance have less impact on underlying soil characteristics. Subsurface moisture-accumulation pathways continued to play a significant role, as shown by the modest relationships that variables like TWI, convergence index, and valley depth maintained with certain Landsat SWIR bands. Although their association with NDVI and EVI was not as significant as in the 0–15 cm layer (Figure 4a), spectral bands maintained high internal correlations. This is expected because vegetation indices measure canopy reflectance rather than underlying soil characteristics. The climatic variable (Worldclim) shows somewhat higher correlations at 15–30 cm than for the topsoil, suggesting that deeper soil layers may respond more reliably to macro-climatic gradients than to surface-reflectance dynamics (Figure 4b).

### 3.3. Feature Selection

**Figure 5:**
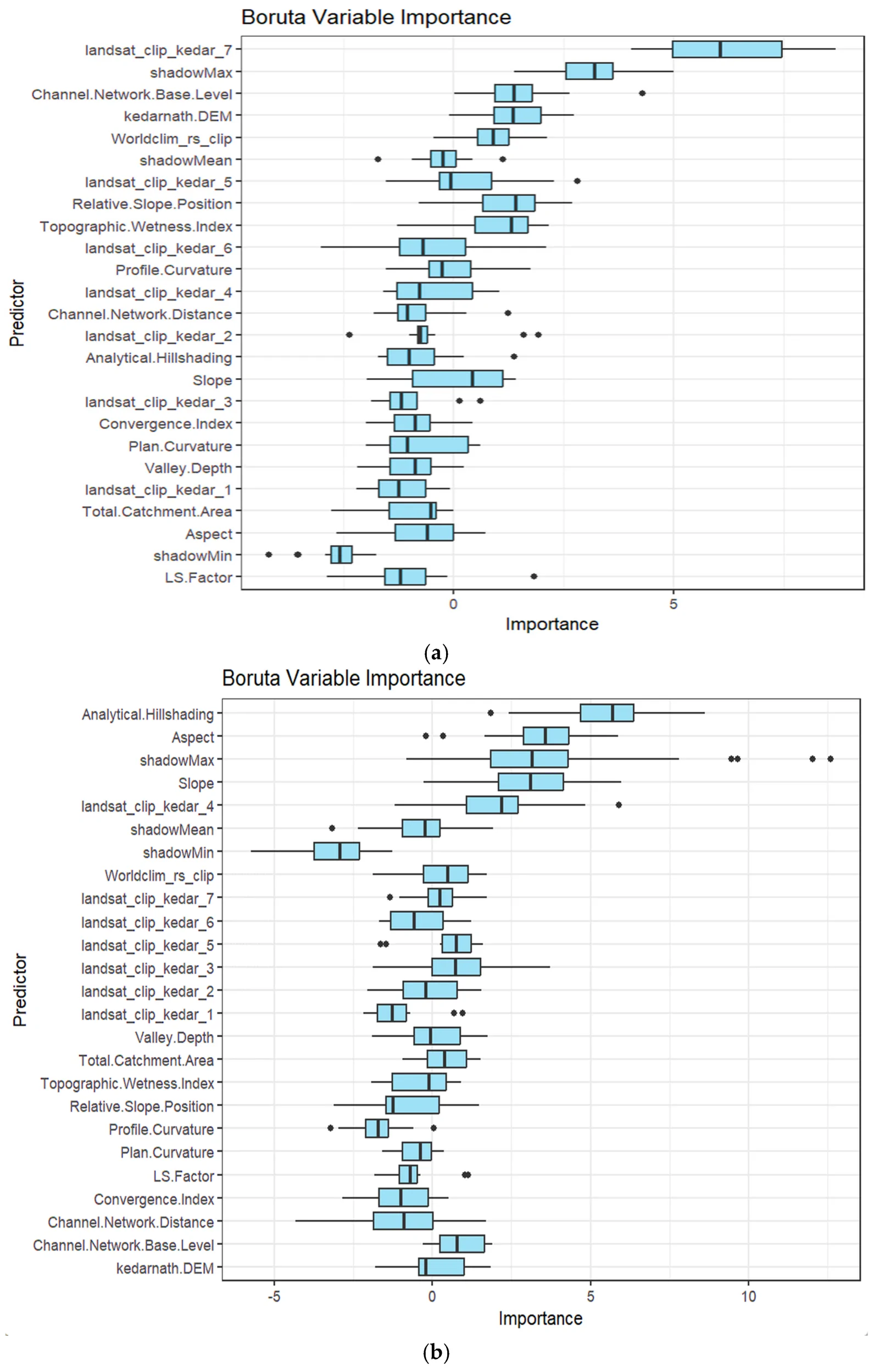
(**a**) Feature-selection-importance graph using the BORUTA algorithm (0–15 cm) and (**b**) feature-selection-importance graph using the BORUTA algorithm (15–30 cm). The black dots in the boxplots represent outlier importance values, corresponding to individual Boruta iterations in which a predictor exhibited unusually high or low importance relative to its interquartile distribution. These points indicate variability in predictor importance and should not be interpreted as statistical significance levels.

Among the 27 covariates considered, several were found to be significant for SOC prediction in the region; other variables were excluded. Significant variables included topographic factors, climatic variables, and spectral bands/indices. The relationship between SOC content and the set of chosen environmental factors was evaluated by computing pair-wise correlation coefficients using Pearson’s approach. The results show modest linear connections between the various variables and SOC, with correlation values ranging from moderate to low. Apart from the clay index, BIO-19, BIO-10, BIO-17, BIO-14, BIO-03, temperature seasonality, slope, TRI, and elevation, most factors showed negative correlations with SOC. The clay index, BIO-19, BIO-10, and TRI show the strongest positive correlation with SOC (0.22), followed by slope (0.2). Many spectral bands/indices show notable negative values, with blue, green, red, and TIR bands showing the largest negative correlation (−0.27), followed by SWIR1 and BI (−0.26). These findings are consistent with results from other investigations in different regions. Complex landscapes of China’s Jilin Province showed similar negative correlation values for SOC with Landsat spectral bands and positive correlations with elevation and slope [30]. SOC was shown to have a negative relationship with temperature and a low positive correlation with elevation. The substantial elevation changes across the research region, the resulting variety in climate and vegetation, and their combined effects are probably the causes of the correlation values observed in this study (Figure 5a,b).

### 3.4. Performance Evaluation of Ml Models

At the surface layer (0–15 cm), there were noticeable performance variations between the three models. RF proved to be the most effective model, with the lowest RMSE (0.35) and the greatest R^2^ (0.62), indicating that the model explained a considerable fraction of SOC variability. The slope value of 2.17 demonstrates a propensity of RF to somewhat overpredict SOC levels; however, predictions were more consistent and closer to the 1:1 line compared to other models. The SVM model performed reasonably in terms of prediction error (RMSE = 0.44), although it had a relatively low R^2^ (0.06), indicating that it captured only a small percentage of SOC variance. The highly negative slope (−1.20) revealed a clear underprediction tendency, where increases in measured SOC were not sufficiently represented in predicted values. At the surface level, the XGBoost model performed poorly. Its extraordinarily high RMSE (3.31) and very poor R^2^ (0.01) showed that the model struggled greatly to define SOC patterns in the surface layer (Table 3).

The slope value near zero (0.07) suggests that the model provided predictions with limited sensitivity to changes in real SOC. At the subsurface layer (15–30 cm), the prediction abilities of most models improved, but RF continued to show superior performance. RF achieved the greatest R^2^ (0.74) and the lowest RMSE (0.33), indicating a stronger predictive relationship at deeper soil depths. Although predictions continued to be consistent with measured SOC, the slope of 2.08 indicated modest overprediction. XGBoost displayed substantial improvement compared to its surface layer performance, with an RMSE of 0.34 and R^2^ of 0.50, demonstrating modest predictive capacity and capturing half of the SOC variability at this depth. The slope (1.46) indicated modest overestimation.

**Figure 6:**
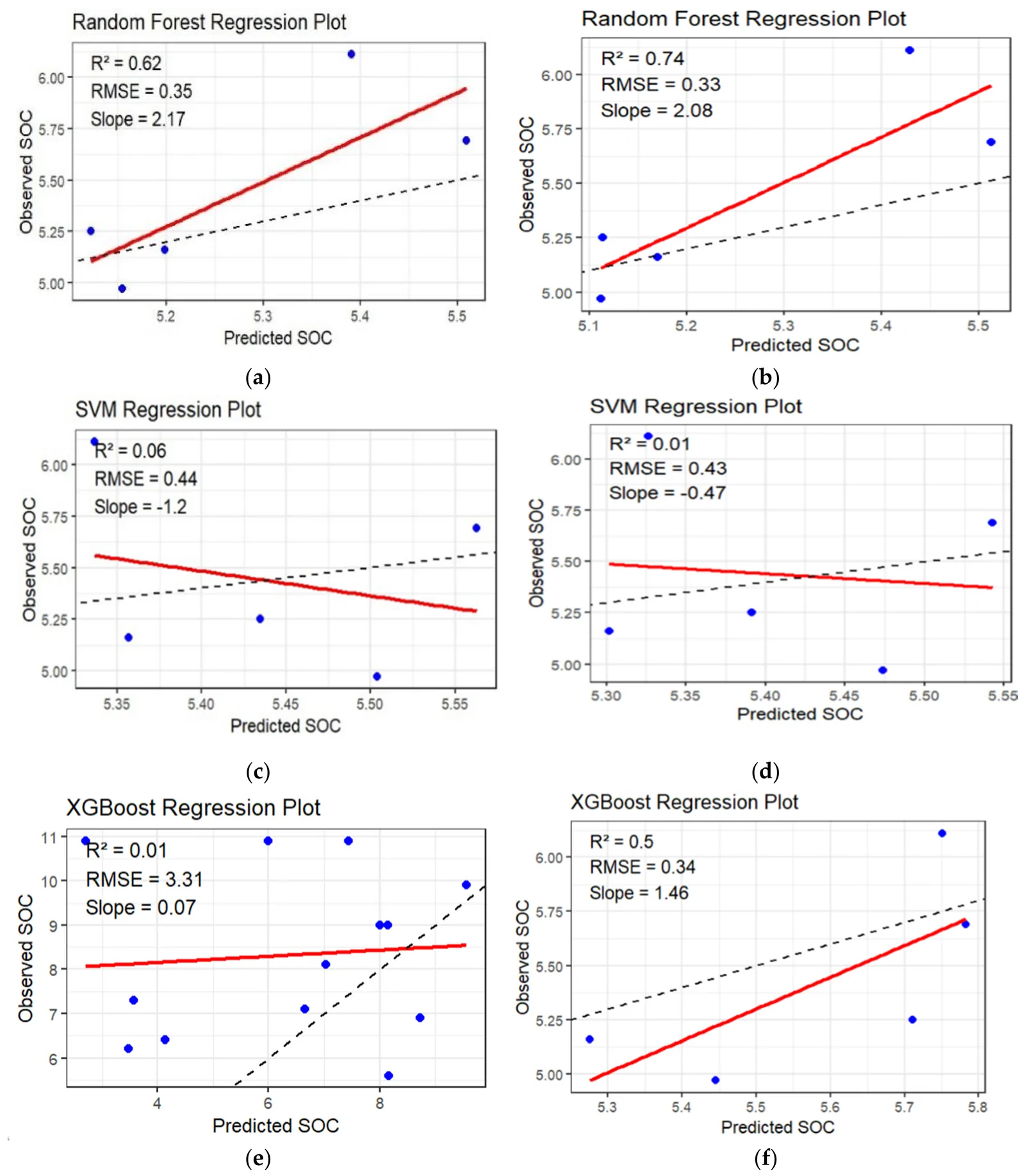
(**a**–**f**). Predicted versus observed soil-organic-carbon values for different models at 0–15 cm (**a**,**c**,**e**) and 15–30 cm (**b**,**d**,**f**) soil depths.

**Figure 7:**
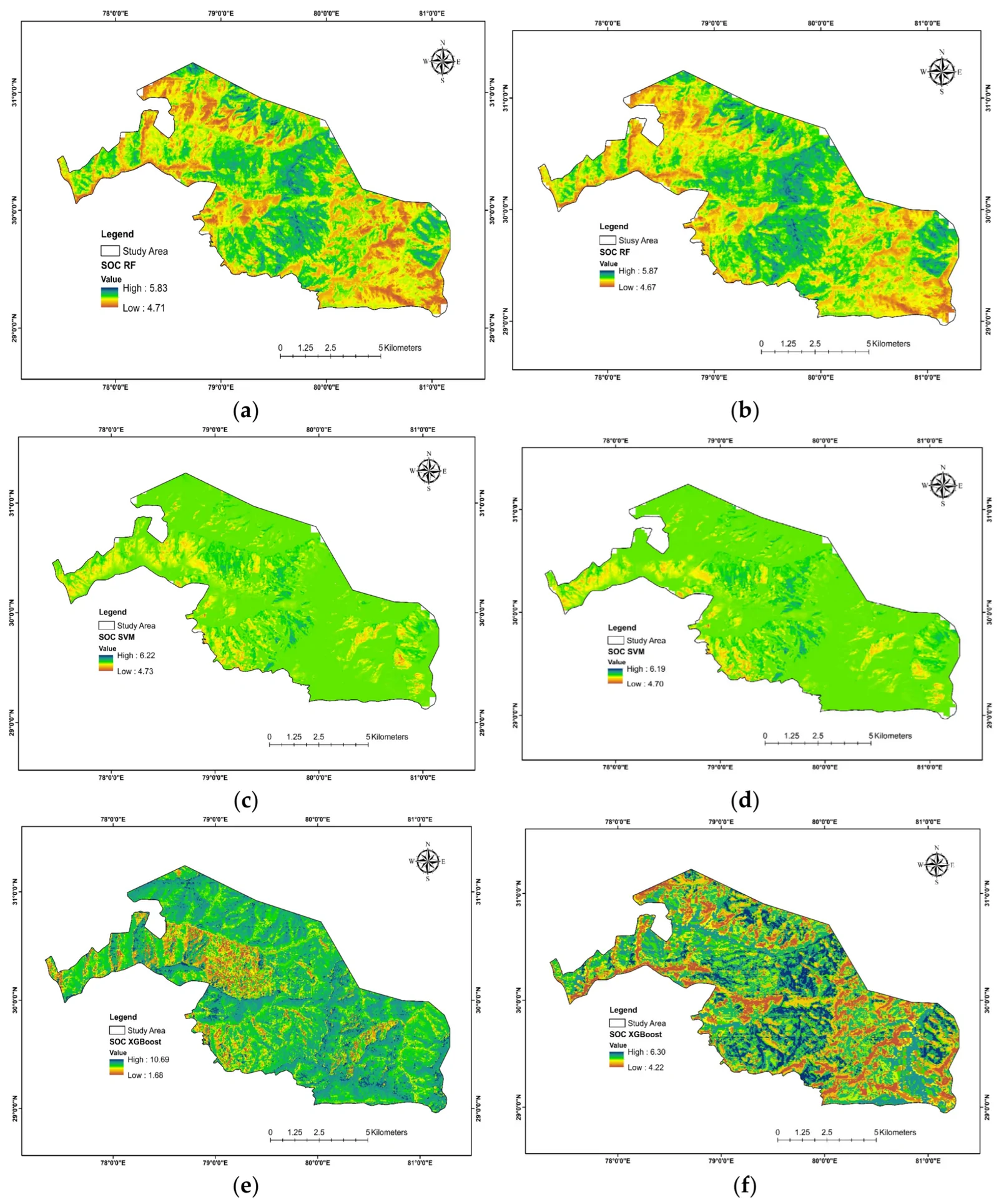
(**a–f**). Spatial prediction of soil organic carbon using machine-learning models: Random Forest at 0–15 cm (**a**) and 15–30 cm (**b**) soil depths; Support Vector Machine at 0–15 cm (**c**) and 15–30 cm (**d**); and XGBoost at 0–15 cm (**e**) and 15–30 cm (**f**).

SVM’s low predictive ability persisted at depth, mirroring its surface-layer performance. The RMSE remained very high (0.43) and R^2^ was extremely low (0.01), demonstrating no explanatory power for SOC distribution at this depth. The slope of −0.47 supported persistent underprediction, indicative of structural challenges in capturing SOC dynamics for Himalayan soils (Figure 6a–f). Overall, RF was the most accurate and reliable model across both depth intervals. RF successfully captured non-linear interactions between SOC and environmental factors in the Himalayan region, as evidenced by consistently higher R^2^ values and lower RMSE values. The minor over-prediction found by slope values > 1 was small and within acceptable bounds for SOC modeling. XGBoost ranked second, with low performance in the surface layer but robust recovery in deeper soils, indicating the depth-specific sensitivity of the model. SVM ranked lowest overall, largely due to its failure to learn SOC variation well, as evidenced by very low R^2^ and negative slope values across both depths (Figure 7a–f).

### 3.5. Prediction-Interval Coverage Probability and Confidence Level of Models

**Figure 8:**
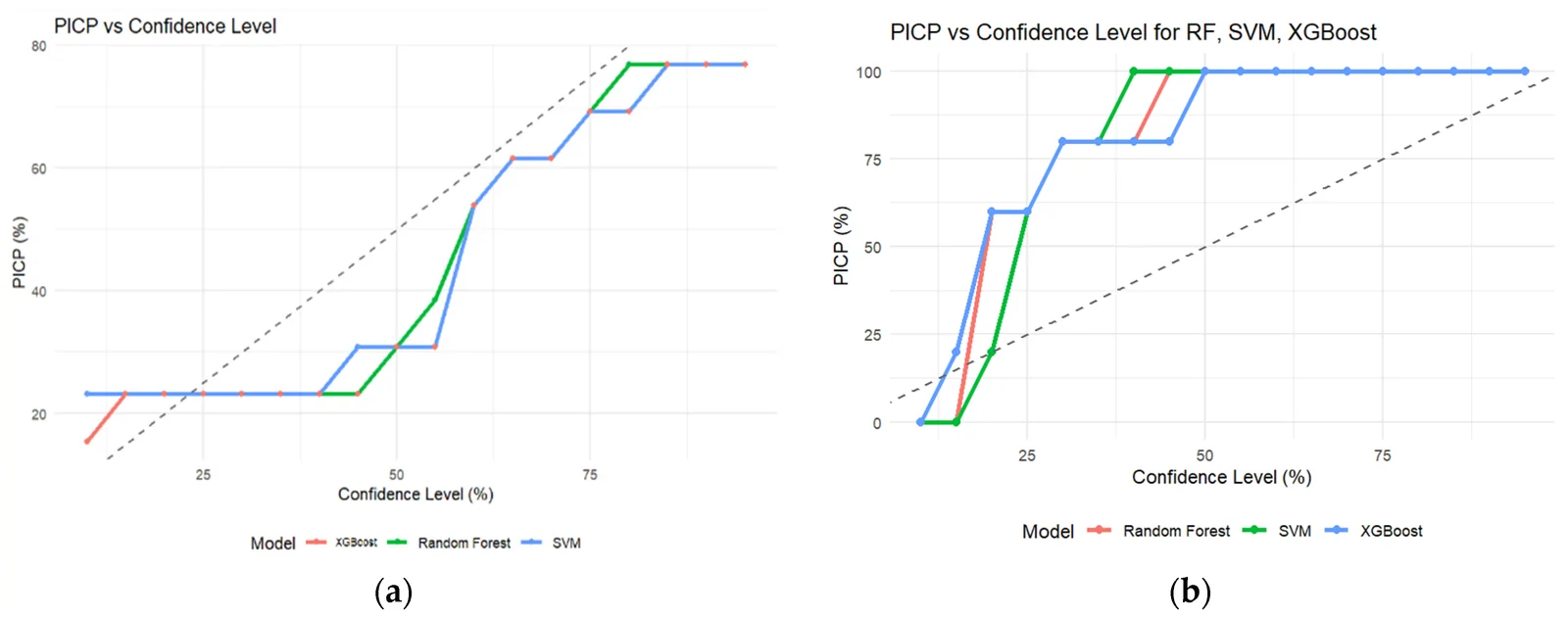
(**a**,**b**). Validation of SOC prediction uncertainty based on the relationship between Prediction-Interval Coverage Probability (PICP) and Confidence Interval (CI) at 0–15 cm (**a**) and 15–30 cm (**b**) soil depths.

The relationship between Prediction-Interval Coverage Probability (PICP) and Confidence Level (CI) at two different soil depths was used to validate prediction uncertainty for SOC estimates. The calibration performance of the three machine-learning models (XGBoost, Random Forest, and SVM) varied significantly across confidence levels. XGBoost had poor calibration; at low-to-moderate confidence levels, it maintained a relatively flat PICP of about 20%, but at higher confidence levels, it showed very little progress. Random Forest and SVM, on the other hand, showed more stable patterns, with PICP values staying low (20–25%) at confidence levels below 50%. Both models, however, eventually reached a plateau of around 75–80% PICP at the highest confidence levels, with a noticeable step-like rise in PICP between 50 and 75% confidence levels. The ideal calibration line (dashed diagonal reference) significantly deviated from all models, demonstrating consistent over-coverage or under-coverage over the confidence spectrum. At deeper soil depths, the model’s performance significantly increased. Tighter prediction intervals were indicated by Random Forest and SVM’s more aggressive PICP responses, which reached near-perfect coverage (≈100% PICP) at a 50% confidence level. Before stabilizing close to 100% PICP, XGBoost showed a more slow but constant increase in PICP, reaching comparable coverage levels at the 75% confidence level. Furthermore, at lower confidence levels, all models showed significant departures from ideal calibration; but, at increasing confidence levels, the alignment with theoretical predictions significantly improved. Overall, these findings suggest that prediction-interval calibration differs considerably between models and soil levels, with deeper soil layers exhibiting better uncertainty quantification performance (Figure 8a,b).

## 4. Discussion

### 4.1. Spatial Dynamics of Soil Organic Carbon

Edaphic characteristics are profoundly influences by geographic, topographic and climatic conditions. In mountains, such conditions change with increasing elevation [9,31]. As elevation increases, temperature and evapotranspiration decrease in these regions. A major controlling factor is temperature, along with elevation, accounted for 73% of the variations in soil organic carbon (SOC) storage [32,33,34]. The pedogenesis, physical, and chemical properties of soil are mainly influenced by elevational variations, which, ultimately, influence the vegetation composition and distribution in mountain ecosystems [35]. In the present study, soil organic carbon (SOC) exhibited a significant declining trend along the elevation gradient in the NW aspect (Figure 3c), and the observations were similar to the earlier reported trends in Himalaya [10,27]. This decline may be attributed to variations in soil texture, reduced litter inputs, and lower decomposition rates, as well as to weakened microbial activity under the low-temperature conditions at higher elevations [36,37,38]. Also, at this particular site (NW aspect), the vegetation composition at higher elevations was quite unique (dominant species- *Abies spectabilies* and krummholz of *Rhododendron campanulatum*) as compared to the NE (dominant species- *Quercus semecarpifolia* and *R. arboreum*) and SW aspects (dominant species- *R. arboretum* and *Q. semecarpifolia*) [39], along with there being a high level of human interference as, at this site/aspect, a pilgrim route to the Tritiyakedar temple Tungnath is present. Meanwhile, in the NE and SW aspects, the results were quite different to those for the NW aspect; in the NE aspect, the upper depth showed a significant increase in SOC (Figure 3b), which might be due to differences in microclimate conditions, habitat and vegetation compositions. Our study also indicates that nutrient concentrations, i.e., SOC decreases with increase in soil depth in two major aspects (NE and NW), were primarily due to the decomposition of organic matter that occurs in the uppermost layers [7,40]. The soil-organic-carbon minimum and maximum ranges are comparable with the reported ranges for the western Himalaya [7,27,40,41], while the minimum and maximum ranges are greater than the reported ranges for the western Himalaya [42,43]. The observed SOC trends highlight the critical role of aspect-dependent microclimates. In the North-East (NE) aspect, SOC significantly increases with elevation because these slopes receive less direct sunlight, resulting in colder and more humid conditions that limit microbial decomposition and facilitate organic carbon accumulation [27]. Conversely, the South-West (SW) aspect exhibits no significant elevational trend. SW slopes receive higher solar radiation, creating warmer, drier environments that accelerate microbial breakdown and soil respiration. Additionally, reduced moisture limits plant growth and fresh organic inputs, effectively counteracting the typical high-altitude accumulation of SOC on these slopes [40].

### 4.2. Soil Organic Carbon Along Elevational Gradient Across Aspects

In mountainous areas, temperature and evapotranspiration often drop with elevation. Depending on the soil’s structure, organic matter content, and other properties, this temperature drop usually leads to more soil moisture for a given quantity of precipitation. Increased soil organic matter and higher rates of snowmelt due to climate change may be partially responsible for the rise in soil moisture. Organic matter-rich soils hold onto more water [10,44] and higher altitudes seem to accumulate more soil organic matter due to a decrease in decomposition rate. These results are consistent with what we found in the NE aspect, where SOC dramatically rose with altitude at both soil depths [45,46].

The disparity in reaction between features illustrates how crucial microclimatic variation is in regulating the dynamics of soil organic carbon. Because they get less direct sunlight than south-west and north-west slopes, northeast-facing slopes are consistently colder and more humid [47]. Organic carbon build-up is made possible by these favorable moisture conditions as well as the lower temperatures that restrict microbial breakdown. According to [48], aspect influences elevational trends since the observed rise in SOC with elevation in the NE aspect was substantially larger than in other aspects (R^2^ = 0.29 for upper soil and R^2^ = 0.21 for lower soil). On the other hand, because of increased evapotranspiration and exposure to more solar radiation, south-facing slopes (SW aspect) are often warmer and drier.

These circumstances may limit organic matter inputs and increase carbon loss through respiration because they speed up the breakdown of organic matter and decrease the amount of moisture available for plant development [49]. This enhanced carbon cycling regime is reflected in the lower elevational trends in SOC on south-facing slopes. Slopes that face northwest have intermediate traits, with moderate solar radiation and intermediate moisture regimes resulting from their orientation.

Topographic orientation heavily influences soil organic carbon (SOC) dynamics through distinct microclimatic and localized factors. The North-East (NE) slopes receive less direct solar radiation, resulting in consistently colder and more humid microclimates that limit microbial breakdown and slow decomposition, thereby facilitating a significant increase in SOC accumulation at higher elevations [47,48,49]. In contrast, the South-West (SW) slopes face direct solar exposure, creating warmer and drier conditions with elevated evapotranspiration. This microclimate accelerates microbial breakdown and soil respiration while limiting continuous plant development and fresh organic inputs, ultimately preventing high-altitude SOC accumulation [47]. Meanwhile, the North-West (NW) aspect exhibits intermediate solar radiation and moisture regimes, yet experiences significant SOC declines as elevation increases. This distinct trend in the NW aspect is driven by a combination of unique high-elevation vegetation, such as Abies spectabilis and *Rhododendron campanulatum*, alongside localized human disturbances like the Tungnath pilgrim route [39].

### 4.3. Depth-Dependent Patterns and Implications for Carbon Cycling

The concentrated nature of organic matter in surface soils is shown by the notable variations in SOC content across all features between higher (0–15 cm) and lower (15–30 cm) depths. This pattern is typical of Himalayan alpine ecosystems, where litter build-up and root biomass concentrate organic matter inputs from plants near the soil’s surface. Higher SOC concentrations were consistently found at deeper soil depths, which is indicative of the principal zone of microbial activity and litter breakdown [50]. Reduced organic inputs, decreased biological activity, and, perhaps, increased microbial digestion of organic compounds are the causes of the lower SOC concentration in subsurface layers (15–30 cm). However, because of the protection provided by mineral matrices and the decreased availability of oxygen, the stability of organic molecules rises with depth, allowing for the longer-term preservation of organic carbon in deeper strata [51]. Since correct carbon sequestration estimates necessitate sampling at several depths and accounts for the non-linear distribution of carbon with depth, these depth-dependent patterns have important implications for estimation of the region’s carbon stocks [52].

### 4.4. Environmental Correlates and Machine-Learning Predictions

According to the correlation study, elevation and terrain-based indices (TRI, RSP, slope, and TWI) are the main factors influencing the distribution of SOC at the surface layer [53]. The microclimate, soil moisture redistribution, plant patterns, and erosion dynamics are all captured by these factors. The combined impacts of temperature gradient, moisture availability, and plant type changes throughout the altitudinal gradient are reflected in the moderately positive correlation between elevation and SOC (r = 0.2) and between slope and SOC (r = 0.2) [54,55]. It is interesting to note that a number of spectral variables, including brightness indices and Landsat bands, had negative relationships with SOC. This surprising result could be explained by the fact that forested areas with high SOC have lower spectral reflectance because of dense vegetation, whereas areas with higher visible reflectance, correspond to exposed, sparsely vegetated areas with low organic matter content. In line with the anticipated association between vegetation productivity and organic matter inputs, vegetation indices (NDVI and EVI) demonstrated positive interactions with SOC. The modest relationships between SOC and bioclimatic variables (BIO-10, BIO-19, BIO-3, and BIO-4) suggest that the dynamics of organic carbon are influenced by the seasonality of temperature and precipitation. There were positive associations between the mean temperature of the hottest quarter and the precipitation of the coldest quarter, indicating that SOC build-up is supported by climatic conditions that both promote plant growth and decrease breakdown.

### 4.5. Machine-Learning-Model Performance: Implications for Digital Soil Mapping

In this Himalayan study, the Random Forest approach outperformed SVM and XGBoost for SOC prediction, which has various significant ramifications. Random Forest is especially well-suited for complicated mountainous terrain, where soil formation is regulated by several interacting factors, because of its capacity to capture nonlinear correlations and interaction effects between various environmental variables [56]. Given the intrinsic spatial complexity and variability of alpine ecosystems, the model explains 62% and 74% of the variance in SOC, respectively, according to the constant R^2^ values of 0.62 and 0.74 at the two depths [57]. While effectively reflecting the general trend, the little overprediction by RF (slopes > 1) indicates a tiny propensity to overestimate SOC values in mid-range forecasts. The model’s usefulness for spatial prediction and carbon-stock assessment is not significantly harmed by this little distortion. Support vector machines have difficulty capturing the intricate, nonlinear linkages present in soil-environmental-variable interactions in this terrain, as seen by the considerable underprediction by SVM at both depths [58]. In this instance, SVM’s linear separability in feature-space assumption is broken, resulting in poor generalization to new data. Gradient-boosting techniques are good in terms of learning depth-specific SOC patterns, but they may overfit local variability at the surface layer, according to XGBoost intermediate performance, especially the significant improvement from surface to subsurface depths. The improved performance at 15–30 cm depth (R^2^ = 0.50) compared to 0–15 cm (R^2^ = 0.01) reflects XGBoost’s ability to find generalizable patterns when subsurface conditions are more uniform and less affected by local disturbances [59].

### 4.6. Spatial Patterns and Implications for Ecosystem Management

Coherent patterns in the distribution of SOC that are compatible with established restrictions on soil formation in the area may be seen in the spatial prediction maps produced by RF. The combined impacts of lower temperatures, increased moisture availability, and denser plant cover in these colder regions are reflected in higher SOC concentrations in the north and northeast [60]. Increased decomposition and, perhaps, decreased plant productivity in warmer, drier microsites are reflected in reduced SOC in exposed southern and southwestern regions. For various management and research purposes, these geographically precise SOC maps offer baseline data that is essential: (1) carbon accounting and the possibility of sequestering carbon in Himalayan forests under climate-change scenarios; (2) determining low-SOC-sensitive regions that would need conservation measures to preserve soil health; (3) focusing restoration efforts on degraded soil regions; (4) evaluating the potential effects of treeline migration under climate change on regional carbon stocks; and (5) providing information for land-use policy choices in the Kedarnath Wildlife Sanctuary and surrounding areas [61]. The depth-wise comparison showing higher SOC in surface layers and progressive decline with depth is consistent with established pedological principles. The smoother spatial patterns at subsurface depths indicate less influence of local-scale disturbances and disturbances (grazing, litter raking, and erosion) that primarily affect surface layers. This suggests that subsurface SOC stocks may be more stable and less vulnerable to immediate land-use impacts, though they remain sensitive to longer-term climate-driven changes in vegetation and decomposition rates.

Soil organic carbon (SOC) is a foundational indicator that dictates ecosystem resilience and vegetation dynamics at the timberline as robust concentrations improve soil structure, enhance water retention, and regulate the nutrient cycling critical for seedling recruitment during climate-induced treeline shifts. Maintaining these SOC pools is essential for broader socio-ecological resilience; they function as a primary Nature-based Solution (NbS) for biodiversity conservation and a means for ensuring that these fragile mountain transition zones achieve long-term land-degradation neutrality [62]. To operationalize these data in conservation planning, high-resolution digital soil mapping (DSM) can pinpoint low-SOC, sensitive microsites to direct immediate, targeted restoration, while baseline spatial maps provide a mechanism for monitoring treeline migrations and quantifying regional carbon sequestration. Furthermore, translating depth-stratified SOC predictions into practical Sustainable Land Management (SLM) and capacity-building frameworks for agricultural and forestry practitioners ensures that small-scale forestry practices and land-use policies within protected areas, such as the Kedarnath Wildlife Sanctuary, are empirically grounded [20].

### 4.7. Comparison with Global Studies

The R^2^ values obtained for RF (0.62 for 0–15 cm and 0.74 for 15–30 cm) compare favorably with global studies of SOC mapping using machine learning. Ref. [63] reported an R^2^ of 0.62 for RF when mapping plough-layer SOC in western Iran, which is directly comparable to our surface layer results. While their SVM and XGBoost models performed better than those in the current study, the consistent superior performance of RF across diverse ecosystems and regions underlines its broad applicability for SOC prediction across varied environmental contexts [64]. The higher model accuracy at 15–30 cm depth in our study may be attributed to less variability in subsurface SOC distributions compared to top layers, where land use, plant cover variation, erosion, and microclimatic variability exert larger effects. This depth-dependent model’s performance has important implications: models trained primarily on surface data may not accurately predict SOC at depth, highlighting the need for depth-stratified modeling approaches in future studies [65].

## 5. Conclusions

This comprehensive study of soil organic carbon across elevational and aspect gradients in the Himalayan timberline ecotone reveals the critical role of microclimate and topography in controlling soil-carbon dynamics. The significant increase in SOC with elevation in cooler, north-facing aspects contrasts sharply with minimal or negative trends in warmer, south-facing aspects, demonstrating how fine-scale topographic variation generates substantial heterogeneity in soil-carbon stocks across short distances. The superior performance of Random Forest for SOC prediction (R^2^ = 0.62–0.74) compared to SVM and XGBoost establishes machine-learning-based digital soil mapping as a viable tool for generating high-resolution SOC maps across complex Himalayan terrain. These maps capture ecologically meaningful spatial patterns and can be used to quantify baseline carbon stocks against which future climate-driven changes can be measured. These findings are crucial for understanding soil-system responses under continuous global warming and for directing conservation and land-management strategies in high-altitude Himalayan forests that provide critical ecosystem services including water supply, carbon sequestration, and species habitat. Understanding these findings, future research should incorporate multi-seasonal sampling to capture the temporal dynamics of soil organic carbon. Utilizing higher-resolution environmental covariates will better resolve fine-scale microclimatic and topographical effects at the plot level. Additionally, subsequent studies should validate the transferability of these machine-learning models across broader, geomorphologically diverse Himalayan regions and adopt depth-stratified modeling approaches to improve subsurface predictions. Finally, integrating unmeasured pedological and biological drivers could further enhance model accuracy beyond current capabilities.

## 6. Limitations of the Study

The modest sample size of 12 plots per 100 m elevation belt across three aspects may limit statistical power for detecting fine-scale variations. Furthermore, single-season sampling (April to mid-June) precludes capturing temporal SOC dynamics, while the 90 m resolution of environmental covariates could mask important localized microclimatic effects. The geographic focus on a single protected area (Kedarnath Wildlife Sanctuary) also means the transferability of these models to other Himalayan regions remains untested. Finally, although the Random Forest model demonstrated strong predictive capability, it explained only 62–74% of the variance, suggesting that unmeasured pedological or biological factors continue to influence SOC distribution.

## Figures and Tables

**Table 1:** Different environmental covariates used in the models for prediction of soil depth.

Soil Forming Factor	Predictor	Abbreviation	Resolution
Relief	Elevation (m)	Elev	90 m
	Slope	Slp	90 m
	Aspect	Asp	90 m
	Topographic Positioning Index	TPI	90 m
	Topographic Ruggedness Index	TRI	90 m
	Topographic Wetness Index	TWI	90 m
	LS-factor	LSF	90 m
	Channel Network Base Level	CNBL	90 m
	Multi-resolution Index of Valley Bottom Flatness	MRVBF	90 m
	Multi-resolution Ridge Top Flatness	MRRTF	90 m
	Relative Slope Position	RSP	90 m
	Valley Depth	VD	90 m
	Plan Curvature	PlaC	90 m
	Profile Curvature	PrC	90 m
	Total Catchment Area	TCA	90 m
Vegetation	Normalized Difference Vegetation Index	NDVI	250 m _16 days
	Enhanced Vegetation Index	EVI	250 m _16 days
Climate	Annual Mean Temperature (°C)	BIO1	1 km
	Mean Diurnal Range (Mean of monthly (max temp–min temp)) (°C)	BIO2	1 km
	Isothermality (Bio_2/Bio_7) (×100)	BIO3	1 km
	Temperature Seasonality (standard deviation × 100) (°C)	BIO4	1 km
	Max Temperature of Warmest Month (°C)	BIO5	1 km
	Min Temperature of Coldest Month (°C)	BIO6	1 km
	Temperature Annual Range (Bio_5-Bio_6) (°C)	BIO7	1 km
	Mean Temperature of Wettest Quarter (°C)	BIO8	1 km
	Mean Temperature of Driest Quarter (°C)	BIO9	1 km
	Mean Temperature of Warmest Quarter (°C)	BIO10	1 km
	Mean Temperature of Coldest Quarter (°C)	BIO11	1 km
	Annual Precipitation (mm)	BIO12	1 km
	Precipitation of Wettest Month (mm)	BIO13	1 km
	Precipitation of Driest Month (mm)	BIO14	1 km
	Precipitation Seasonality (Coefficient of Variation) (mm)	BIO15	1 km
	Precipitation of Wettest Quarter (mm)	BIO16	1 km
	Precipitation of Driest Quarter (mm)	BIO17	1 km
	Precipitation of Warmest Quarter (mm)	BIO18	1 km
	Precipitation of Coldest Quarter (mm)	BIO19	1 km

**Table 2:** Summary of soil organic carbon along elevation gradient across three representative aspects.

Elevation (m asl.)	South-West Aspect		North-East Aspect		North-West Aspect	
	0–15 cm	15–30 cm	0–15 cm	15–30 cm	0–15 cm	15–30 cm
2100–2200	6.06 ± 0.20	4.62 ± 0.30	5.28 ± 0.19	4.55 ± 0.68	7.15 ± 0.05	6.82 ± 0.29
2200–2300	4.47 ± 0.87	3.87 ± 1.33	5.64 ± 0.72	4.97 ± 0.36	7.32 ± 0.47	5.73 ± 0.56
2300–2400	6.60 ± 0.57	5.59 ± 0.43	6.49 ± 0.17	6.32 ± 0.94	7.16 ± 0.18	6.80 ± 0.38
2400–2500	5.86 ± 0.14	5.41 ± 0.48	5.62 ± 1.05	5.25 ± 0.64	6.05 ± 0.15	5.62 ± 0.14
2500–2600	6.36 ± 0.15	4.80 ± 0.37	6.35 ± 0.15	5.16 ± 0.38	5.32 ± 0.54	3.53 ± 1.27
2600–2700	4.28 ± 0.34	3.75 ± 0.12	6.34 ± 0.29	4.26 ± 0.39	5.21 ± 0.56	4.32 ± 1.03
2700–2800	4.39 ± 0.31	4.31 ± 0.26	6.10 ± 0.81	5.32 ± 0.35	4.76 ± 0.26	4.54 ± 0.23
2800–2900	4.94 ± 0.72	4.45 ± 0.13	6.74 ± 0.26	6.11 ± 0.07	4.37 ± 0.06	4.28 ± 0.12
2900–3000	6.53 ± 0.68	5.45 ± 0.95	6.65 ± 0.50	6.14 ± 0.38	4.22 ± 0.41	3.75 ± 0.34
3000–3100	5.86 ± 0.96	4.99 ± 0.57	6.55 ± 0.29	5.73 ± 0.34	3.97 ± 0.24	3.33 ± 0.18
3100–3200	5.81 ± 0.13	4.95 ± 0.45	6.27 ± 0.51	5.69 ± 0.33	3.61 ± 0.23	2.69 ± 0.23
3200–3300	4.84 ± 1.22	4.00 ± 1.18	000	000	3.06 ± 0.11	1.75 ± 0.26

**Table 3:** Comparison of performance of different models for prediction of soil depth in the Kedar wildlife sanctuary in India.

Soil Depth	0–15 cm			15–30 cm		
Models	RMSE	R^2^	Slope	RMSE	R^2^	Slope
RF	0.35	0.62	2.17	0.33	0.74	2.08
SVM	0.44	0.06	−1.2	0.43	0.01	−0.47
XGBoost	3.31	0.01	0.07	0.34	0.50	1.46

Abbreviations used: RMSE: root mean square error and R^2^: coefficient of determination.

## Data Availability

The original contributions presented in this study are included in the article. Further inquiries can be directed to the corresponding author.

## References

[1] Singh S.P., Bhattacharyya A., Mittal A., Pandey A., Tewari A., Latwal A., David B., Adhikari B.S., Kumar D., Negi G.C.S. (2021). Indian Himalayan timberline ecotone in response to climate change–initial findings. Curr. Sci..

[2] El-Ramady H., Brevik E.C., Abowaly M., Ali R., Saad Moghanm F., Gharib M.S., Mansour H., Fawzy Z.F., Prokisch J. (2024). Soil degradation under a changing climate: Management from traditional to nano-approaches. Egypt. J. Soil Sci..

[3] Barman T., Paul S., Samant S.S., Pangtey D., Chauhan A., Thakur S., Tewari L.M., Lata S. (2025). Population ecology and habitat suitability modelling of *Quercus leucotrichophora* A. Camus in relation to climate change in the Himalaya. Proc. Indian Natl. Sci. Acad..

[4] Mishra G., Das P.K., Borah R., Dutta A. (2017). Investigation of phytosociological parameters and physico-chemical properties of soil in tropical semi-evergreen forests of Eastern Himalaya. J. For. Res..

[5] Tripathi S.K., Chanda R., Ao A., Moirangthem B., Chauhan S., Mizo L., Singh S.S., Singh N.S., Upadhyay K.K., Vanlalfakawma D.C. (2025). Elevation and management-induced vegetation and soil carbon shift in Eastern Himalayan forests: Advancing nature-based sustainability solutions (NbS). Environ. Sustain. Indic..

[6] Kumar S., Khanduri V.P. (2024). Impact of climate change on the Himalayan alpine treeline vegetation. Heliyon.

[7] Kewlani P., Negi V.S., Bhatt I.D., Rawal R.S., Nandi S.K. (2021). Soil nutrients concentration along altitudinal gradients in Indian Western Himalaya. Scand. J. For. Res..

[8] Mishra G., Giri K., Nath A.J., Francaviglia R. (2023). Soil Carbon Dynamics in Indian Himalayan Region.

[9] Mangral Z.A., Islam S.U., Tariq L., Kaur S., Ahmad R., Malik A.H., Goel S., Baishya R., Barik S.K., Dar T.U.H. (2023). Altitudinal gradient drives significant changes in soil physico-chemical and eco-physiological properties of *Rhododendron anthopogon*: A case study from Himalaya. Front. For. Glob. Change.

[10] Müller M., Oelmann Y., Schickhoff U., Böhner J., Scholten T. (2017). Himalayan treeline soil and foliar C:N:P stoichiometry indicate nutrient shortage with elevation. Geoderma.

[11] Mishra G., Sharma A., Barman T., Moharana P.C., Suleymanov A. (2026). Machine learning-based soil depth estimation from the diverse landscapes of Nagaland, North-Eastern Himalayas. J. Mt. Sci..

[12] Sharma A., Mishra G., Kotiyal P.B., Pangtey D., Barman T. (2026). Mapping land use transitions in Sambhal district using spatiotemporal analysis and artificial neural networks. Discov. Geosci..

[13] Hengl T., MacMillan R.A. (2018). Predictive Soil Mapping with R.

[14] Suleymanov R., Yurkevich M., Bakhmet O., Popova T., Kungurtsev A., Zakirov D., Novikova S., Kovalenko V., Fedorenko Y., Suleymanov A. (2025). Interpretable machine learning and remote sensing data reveal soil biogeochemistry patterns in agricultural systems. Land.

[15] Moharana P.C., Mishra G., Keshavarzi A., Suleymanov A. (2026). Harnessing Digital Mapping for Soil Resource Monitoring and Assessment.

[16] Dasila K., Rawal R., Barman T., Samant S.S., Pandey A., Pande V. (2024). Influence of edaphic factors on distribution and condition of Himalayan silver birch (*Betula utilis* D. Don) communities in the northwestern Indian Himalayas. J. Mt. Sci..

[17] Sharma R., Singh S., Huria A., Chauhan A., Barman T. (2024). Sustainable land management practices for healthy ecosystem services: A cornerstone of biodiversity sustenance in the Indian Northwestern Himalaya. Sustainable Land Management in India.

[18] Paul S., Lata S., Barman T. (2023). Habitat distribution modeling of the *Pinus gerardiana* under projected climate change in the North-Western Himalaya, India. Landsc. Ecol. Eng..

[19] Barman T., Samant S.S., Singh A., Tewari L.M. (2021). Population assessment, indigenous uses, and threat status of orchids in ban oak (*Quercus oblongata* D. Don) forests of Himachal Pradesh, Northwestern Himalaya. J. Orchid Soc. India.

[20] Rawal R.S., Rawal R., Rawat B., Negi V.S., Pathak R. (2018). Plant species diversity and rarity patterns along altitude range covering treeline ecotone in Uttarakhand: Conservation implications. Trop. Ecol..

[21] Gairola S. (2008). Forest Vegetation Patterns Along an Altitudinal Gradient in Sub-alpine Zone of West Himalaya, India. Afr. J. Plant Sci..

[22] Rai I.D., Singh G., Pandey A., Rawat G.S. (2019). Ecology of treeline vegetation in western Himalaya: Anthropogenic and climatic influences. Tropical Ecosystems: Structure, Functions and Challenges in the Face of Global Change.

[23] Rawal R., Negi V.S., Tewari L.M. (2023). Forest dynamics along altitudinal gradient covering treeline ecotone of Indian Western Himalaya. Biologia.

[24] Rawal R., Negi V.S., Bhatt I.D., Tiwari L.M. (2024). Rarities pattern of vascular plants in the high-altitude forests of Indian western Himalaya: Conservation implications. J. Nat. Conserv..

[25] Rawal R., Dasila K., Kishor K., Tewari L.M. (2025). Pattern of forest structure and species regeneration along with elevation gradients and aspects in evergreen oak forest belt of the Western Himalaya. Discov. Plants.

[26] Sharma C.M., Gairola S., Ghildiyal S.K., Suyal S. (2010). Physical properties of soils in relation to forest composition in moist temperate valley slopes of the Central Western Himalaya. J. For. Sci..

[27] Rawal R., Dasila K., Negi V.S., Tewari L.M. (2026). Elevation-driven influences on soil nutrient dynamics and vegetation structure in temperate subalpine forests of the Western Himalaya. Sci. Rep..

[28] Walkley A., Black I.A. (1934). An examination of the Degtjareff method for determining soil organic matter, and a proposed modification of the chromic acid titration method. Soil Sci..

[29] Hijmans R.J., Cameron S.E., Parra J.L., Jones P.G., Jarvis A. (2005). Very high-resolution interpolated climate surfaces for global land areas. Int. J. Climatol. A J. R. Meteorol. Soc..

[30] Zhang Y., Shen H., Gao Q., Zhao L. (2020). Estimating soil organic carbon and pH in Jilin Province using Landsat and ancillary data. Soil Sci. Soc. Am. J..

[31] Chauvier Y., Thuiller W., Brun P., Lavergne S., Descombes P., Karger D.N., Pellissier L., Randin C.F., Vittoz P., Zimmermann N.E. (2021). Influence of climate, soil, and land cover on plant species distribution in the European Alps. Ecol. Monogr..

[32] Tashi S., Singh B., Keitel C., Adams M. (2016). Soil carbon and nitrogen stocks in forests along an altitudinal gradient in the eastern Himalayas and a meta-analysis of global data. Glob. Change Biol..

[33] Ahirwal J., Nath A., Brahma B., Deb S., Sahoo U.K., Nath A.J. (2021). Patterns and driving factors of biomass carbon and soil organic carbon stock in the Indian Himalayan region. Sci. Total Environ..

[34] Thakur S., Negi V.S., Dhyani R., Bhatt I.D., Yadava A.K. (2022). Influence of environmental factors on tree species diversity and composition in the Indian western Himalaya. For. Ecol. Manag..

[35] Badía D., Ruiz A., Girona A., Martí C., Casanova J., Ibarra P., Zufiaurre R. (2016). The influence of elevation on soil properties and forest litter in the Siliceous Moncayo Massif, SW Europe. J. Mt. Sci..

[36] Saeed S., Barozai M.Y.K., Ahmad A., Shah S.H. (2014). Impact of altitude on soil physical and chemical properties in Sra Ghurgai (Takatu mountain range) Quetta, Balochistan. Int. J. Sci. Eng. Res..

[37] Manral V., Bargali K., Bargali S.S., Karki H., Chaturvedi R.K. (2023). Seasonal dynamics of soil microbial biomass C, N and P along an altitudinal gradient in central Himalaya, India. Sustainability.

[38] Vlček V., Juřička D., Valtera M., Dvořáčková H., Štulc V., Bednaříková M., Nývlt D., Láska K., Kňažek M., Enev V. (2024). Soil organic matter interactions along the elevation gradient of the James Ross Island (Antarctica). Soil.

[39] Kumar V., Barman T., Pangtey D., Bhondge S.W., Sharma R. (2026). Ecological assessment and germination patterns of *Rhododendron arboreum* in the Himalaya for conservation strategies. Discov. Plants.

[40] Gairola S., Sharma C.M., Ghildiyal S.K., Suyal S. (2012). Chemical properties of soils in relation to forest composition in moist temperate valley slopes of Garhwal Himalaya, India. Environmentalist.

[41] Singh S.K., Pandey C.B., Sidhu G.S., Sarkar D., Sagar R. (2011). Concentration and stock of carbon in the soils affected by land uses and climates in the western Himalaya, India. Catena.

[42] Digvijay R., Gopal R., Kumar Y., Ramola G.C. (2020). Soil Chemical Properties of *Cedrus deodara* (Roxb) G. Don. Forest Soil in Garhwal Himalaya, India. J. Tree Sci..

[43] Ramola G.C., Rathod D., Kumar Y., Dhaval P., Kukreti A., Khanduri V.P. (2020). Changes in the Physico-Chemical Properties of Soil in Different Deodarforests of Garhwal Himalaya. J. Plant Dev. Sci..

[44] Liu Y., Cui Z., Xie S., Ma C., Wei Y. (2026). Molecular evidence for depth-dependent microbial transformation of dissolved organic matter into carboxyl-rich alicyclic molecules in coastal marginal seas. Limnol. Oceanogr..

[45] Simon A., Dhendup K., Rai P.B., Gratzer G. (2018). Soil carbon stocks along elevational gradients in Eastern Himalayan Mountain forests. Geoderma Reg..

[46] Barman T., Samant S.S., Tewari L.M., Kanwar N., Singh A., Paul S., Lata S. (2023). Ecological assessment and suitability ranges of Ban oak (*Quercus oblongata* D. Don) in Chamba district, Himalayas: Implications for present and future conservation. Braz. J. Bot..

[47] Tsozué D., Nghonda J.P., Tematio P., Basga S.D. (2019). Changes in soil properties and soil organic carbon stocks along an elevation gradient at Mount Bambouto, Central Africa. Catena.

[48] Li C., Cao Z., Chang J., Zhang Y., Zhu G., Zong N., He Y., Zhang J., Shi P., He N. (2017). Elevational gradient affects functional fractions of soil organic carbon and aggregates stability in a Tibetan alpine meadow. Catena.

[49] Djukic I., Zehetner F., Tatzber M., Gerzabek M.H. (2010). Soil organic-matter stocks and characteristics along an Alpine elevation gradient. J. Plant Nutr. Soil Sci..

[50] Pei J., Li J., Luo Y., Rillig M.C., Smith P., Gao W., Li B., Fang C., Nie M. (2025). Patterns and drivers of soil microbial carbon use efficiency across soil depths in forest ecosystems. Nat. Commun..

[51] Liu Z., Zhou W., Wu X., Xiong X., Li Q., Kang H., Shukla T., Zhang Q., Kang S., Yin X. (2026). Altitudinal patterns of soil organic carbon and its drivers in the mountains of southeastern Tibet. Land Degrad. Dev..

[52] Gray H.J., Keen-Zebert A., Furbish D.J., Tucker G.E., Mahan S.A. (2020). Depth-dependent soil mixing persists across climate zones. Proc. Natl. Acad. Sci. USA.

[53] Liu F., Yang F., Zhao Y.-G., Li D.-C. (2022). Predicting soil depth in a large and complex area using machine learning and environmental correlations. J. Integr. Agric..

[54] Kang Y., Ozdogan M., Zhu X., Ye Z., Hain C., Anderson M. (2020). Comparative assessment of environmental variables and machine learning algorithms for maize yield prediction in the US Midwest. Environ. Res. Lett..

[55] Mishra U., Yeo K., Adhikari K., Riley W.J., Hoffman F.M., Hudson C., Gautam S. (2022). Empirical relationships between environmental factors and soil organic carbon produce comparable prediction accuracy to machine learning. Soil Sci. Soc. Am. J..

[56] Meier M., Souza E.D., Francelino M.R., Fernandes Filho E.I., Schaefer C.E.G.R. (2018). Digital soil mapping using machine learning algorithms in a tropical mountainous area. Rev. Bras. Ciência Solo.

[57] Kumaraperumal R., Pazhanivelan S., Geethalakshmi V., Nivas Raj M., Muthumanickam D., Kaliaperumal R., Shankar V., Nair A.M., Yadav M.K., Tarun Kshatriya T.V. (2022). Comparison of machine learning-based prediction of qualitative and quantitative digital soil-mapping approaches for Eastern Districts of Tamil Nadu, India. Land.

[58] Zhu C., Wei Y., Zhu F., Lu W., Fang Z., Li Z., Pan J. (2022). Digital mapping of soil organic carbon based on machine learning and regression kriging. Sensors.

[59] Zhang X., Xue J., Chen S., Wang N., Shi Z., Huang Y., Zhuo Z. (2022). Digital mapping of soil organic carbon with machine learning in dryland of Northeast and North plain China. Remote Sens..

[60] Wang S., Zhuang Q., Wang Q., Jin X., Han C. (2017). Mapping stocks of soil organic carbon and soil total nitrogen in Liaoning Province of China. Geoderma.

[61] Falahatkar S., Hosseini S.M., Ayoubi S., Salmanmahiny A. (2016). Predicting soil organic carbon density using auxiliary environmental variables in northern Iran. Arch. Agron. Soil Sci..

[62] Barman T., Mishra G., Sowińska-Świerkosz B., Abhilash P.C., Francaviglia R. (2026). Nature-based solutions for environmental sustainability: Enhancing the resilience of mountain ecosystems in the era of climate change. Curr. Opin. Environ. Sustain..

[63] Mahmoudzadeh H., Matinfar H.R., Taghizadeh-Mehrjardi R., Kerry R. (2020). Spatial prediction of soil organic carbon using machine learning techniques in western Iran. Geoderma Reg..

[64] Zeraatpisheh M., Ayoubi S., Jafari A., Tajik S., Finke P. (2019). Digital mapping of soil properties using multiple machine learning in a semi-arid region, central Iran. Geoderma.

[65] Adeniyi O.D., Brenning A., Bernini A., Brenna S., Maerker M. (2023). Digital mapping of soil properties using ensemble machine learning approaches in an agricultural lowland area of Lombardy, Italy. Land.

